# Nucleocapsid protein accumulates in renal tubular epithelium of a post-COVID-19 patient

**DOI:** 10.1128/spectrum.03029-23

**Published:** 2023-11-17

**Authors:** Anita E. Grootemaat, Niek Wiersma, Sanne van der Niet, Irene M. Schimmel, Sandrine Florquin, Eric A. Reits, Sara E. Miller, Nicole N. van der Wel

**Affiliations:** 1 Electron Microscopy Centre Amsterdam, Medical Biology, Amsterdam University Medical Centre AMC, Amsterdam, the Netherlands; 2 Amsterdam Institute for Infection and Immunity, Amsterdam, the Netherlands; 3 Department of Pathology, Amsterdam University Medical Centers (location University of Amsterdam), Amsterdam, the Netherlands; 4 Department of Pathology, Duke University Medical Center, Durham, North Carolina, USA; University of Mississippi Medical Center, Jackson, Mississippi, USA

**Keywords:** SARS-CoV-2, electron microscopy, fluorescent microscopy, pathology

## Abstract

In this study, we investigated whether severe acute respiratory syndrome coronavirus 2 (SARS-CoV-2) viral proteins and virus particles can be detected (i) in post-mortem kidney of fatal coronavirus disease 2019 (COVID-19) patients and (ii) in renal biopsy of patients suffering from kidney failure months after combating a SARS-CoV-2 infection. Using fluorescence microscopy, antibodies against viral proteins [nucleocapsid, membrane (M), non-structural proteins (nsp) 3, 4, and 13] and double-stranded RNA (dsRNA) were validated on SARS-CoV-2-infected Vero cells. dsRNA was detected in lipid-filled electron-lucent (white) compartments in SARS-CoV-2-producing cells. In the kidney of a patient with acute kidney failure, accumulation of SARS-CoV-2 nucleocapsid N protein was detected in tubular epithelium. In contrast, none of the other viral proteins (M, nsp3, 4, 13) nor dsRNA was detected in these regions, suggesting that no viral replication takes place in the kidney of these patients. High-resolution immunoelectron microscopy demonstrated N protein accumulation in Golgi-like structures but absence of intracellular virus particles. The presence of the N protein in Golgi-like structures in kidney of patients suggests that this protein may have an unforeseen role in post-COVID-19 renal complications.

**IMPORTANCE:**

Even though the coronavirus disease 2019 (COVID-19) pandemic is slowly developing into a conventional infectious disease, the long-term effects of severe acute respiratory syndrome coronavirus 2 (SARS-CoV-2) virus infection are still not well understood. One of the problems is that many COVID-19 cases develop acute kidney injuries. Still, it is heavily debated whether SARS-CoV-2 virus enters and actively replicates in kidney tissue and if SARS-CoV-2 virus particles can be detected in kidney during or post-infection. Here, we demonstrated that nucleocapsid N protein was detected in kidney tubular epithelium of patients that already recovered form COVID-19. The presence of the abundantly produced N protein without signs of viral replication could have implications for the recurrence of kidney disease and have a continuing effect on the immune system.

## INTRODUCTION

Since its discovery in December of 2019 (1), the novel coronavirus severe acute respiratory syndrome coronavirus 2 (SARS-CoV-2) has had a profound medical, social, and economic impact on society. Regardless of vaccines alleviating the healthcare system of a number of hospitalized patients, there are still cases of severe and critical coronavirus disease 2019 (COVID-19) cases that require intensive treatment. Despite the main body of research focusing on the pulmonary and immune systems, it has been shown that many other extra-pulmonary tissues are also affected during COVID-19 (2, 3). Of affected tissues, acute kidney injury (AKI) is an established extra-pulmonary complication of COVID-19 (4 – 6), even for children (7). A meta-analysis (8) found that 4.5% of all confirmed COVID-19 cases develop AKI, mostly prevalent in critical COVID-19 cases. More recent studies report the incidence of AKI to be 15%–25% and is specifically high in COVID-19 patients with pre-existing complications (9). Due to the high incidence of kidney damage during COVID-19, understanding precisely how SARS-CoV-2 affects the kidney during COVID-19 can help improve treatment of patients during hospitalization, as well as improve recovery of long-term COVID-19 or post-COVID-19 patients suffering from kidney complications.

As for the origin of AKI during COVID-19, many causal factors have been proposed, including hyper-coagulation, microangiopathy, rhabdomyolysis, endothelial activation, dysregulation of complement, and angiotensin-converting enzyme 2 (ACE2) pathway activation (9, 10). In addition, the direct cytotoxic effect of viral infection has also been suggested as a cause of kidney damage. In kidney, the viral receptor ACE2 is highly expressed by both proximal tubular epithelial cells and parietal epithelial cells (11) which highlights the potential susceptibility of kidney to SARS-CoV-2 infection. Still, it is heavily debated whether SARS-CoV-2 enters or actively replicates in kidney tissue. The presence of SARS-CoV-2 in kidney during COVID-19 has previously been investigated by multiple studies employing RNA *in situ* hybridization, immunohistochemistry (IHC), transmission electron microscopy (TEM), confocal microscopy, quantitative reverse transcription PCR, or RNA assays (11 – 18). In these studies, SARS-CoV-2 proteins or RNA were detected in multiple extra-pulmonary tissues; including liver, spleen, heart, prostate, uterus, colon, kidney, lymph node, and thyroid. However, a pitfall of IHC and RNA assays is that the presence of viral components is proven, but viral particles and replication organelles cannot be directly observed. TEM provides great resolution at high magnifications and enables direct visualization of virus particles and virus-induced membrane structures. Even so, identification of virus with TEM remains challenging, because many ordinary intracellular structures mimic virus particles (19 – 22). Immunoelectron microscopy aids identification of viral particles by enabling detection of viral components in TEM sections, using antiviral antibodies in conjunction with electron dense gold particles (21, 23). Most TEM-based work related to SARS-CoV-2 have been performed on samples embedded in plastic, where immunogold labeling is less efficient. Here, we used gold labeling of ultrathin cryo-sections and electron microscopy of COVID-19 kidney, to determine whether SARS-CoV-2 virus particles and replication complexes are present.

Kidney samples from patients with fatal COVID-19 or post-COVID-19 patients were first screened for viral presence using an antibody against a SARS-CoV-2 protein followed by a fluorescent secondary antibody. We selected antibodies already validated on SARS-CoV-2-infected Vero cells (23). This way, the presence of viral structural proteins like nucleocapsid protein (N) and membrane (M) protein was tested and the presence of the viral replication complex was tested using antibodies against double-stranded RNA (dsRNA) (24, 25), non-structural proteins (nsp) 3, 4, and 13. Using correlative light and electron microscopy (CLEM), dsRNA was observed in lipid-filled structures in infected Vero cells (monkey kidney cell line), demonstrating a link between lipid accumulation and viral replication.

## RESULTS

### SARS-CoV-2 N protein in Vero cells was detected by fluorescence microscopy (FM)

**Fig 1:**
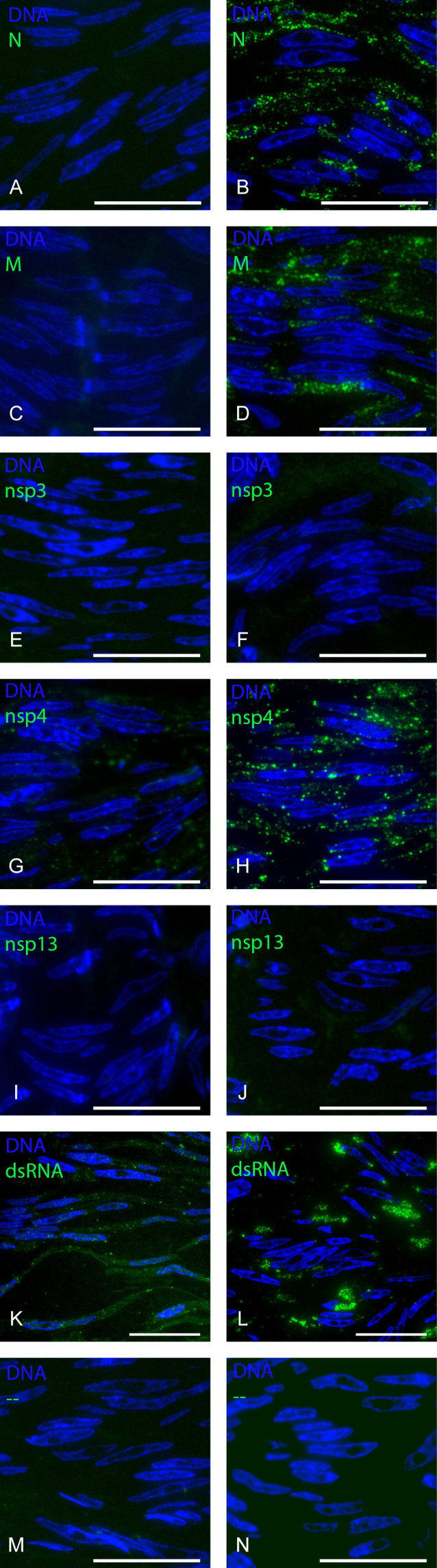
Fluorescence microscopy detection of viral proteins on SARS-CoV-2-infected and uninfected Vero cells. Cryo-sections of 300 nm in thickness from uninfected (left) and 24 h-infected SARS-CoV-2 (right) Vero cells were stained with Hoechst dye to identify nuclei (blue) and primary antibodies followed by secondary antibody labeled with Alexa 488 (green) detecting viral components as follows: (A, B) N protein; (C, D) M protein; (E, F) nsp3; (G, H) nsp4; (I, J) nsp13; and (K, L) dsRNA. As a control (M, N), the primary antibody was omitted and only secondary fluorescent antibody Alexa 488 was used. Scale bar represents 20 µm.

In order to determine which antibodies can be used to screen patient kidney biopsies for regions with viral proteins, we characterized the quality of antibodies that recognize viral proteins on semi-thin (200–300 nm) cryo-sections of SARS-CoV-2-infected Vero cells using FM. The selection of antibodies was based on previous work on glutaraldehyde- and paraformaldehyde-fixed lung tissues and cells (23). Vero cells were infected with SARS-COV-2 and fixed 24 h post-infection, as at this time point, the infection is established, and viral proteins are being produced (26). In order to validate the various SARS-CoV-2-specific antibodies, both uninfected and SARS-CoV-2-infected Vero cells were stained for viral N and M proteins, nsp3, 4, and 13, and dsRNA (Fig. 1). The N and M protein antibodies revealed a dotted, cytoplasmic pattern in the perinuclear region of infected Vero cells, whereas uninfected cells showed homogeneous weak background fluorescence (Fig. 1A through D). The only nsp3 antibody we tested was very weak, even after testing multiple dilutions (1:10–1:1,000) and only stained a few spots per infected cell (Fig. 1E and F). The antibody against nsp4 showed background staining in uninfected cells (Fig. 1G), even at lower concentrations (data not shown). Still, nsp4 staining in infected cells demonstrates a clear dotted pattern spread throughout the whole cell (Fig. 1H). The nsp13 antibody did not specifically stain infected cells at this time point of infection (Fig. 1I and J). The dsRNA labeling in uninfected Vero cells shows a dotted pattern present in the nucleus and cytosol, but in SAR-CoV-2-infected cells, the staining is very distinct in the perinuclear region (Fig. 1K and L). As an extra control, secondary fluorescent antibody was used without a primary antibody (Fig. 1M and N), and no signal was detected for both infected and uninfected cells. Thus, antibodies against N protein, M protein, nsp4, and dsRNA can be used reliably for FM, although there may be some nonspecific background staining for nsp4 and dsRNA.

### SARS-CoV-2 proteins were not observed in kidney from fatal COVID-19 patients

Tissue was obtained from six different COVID-19 patients (patient 1–6 in Table 1). These patients were from the first wave of infections in The Netherlands (27), and lung tissue had already been analyzed and found to be positive for viral proteins using immunoelectron microscopy (IEM) (23). Kidney blocks were fixed identically as the successfully analyzed lung and processed for IEM as soon as possible, then embedded in gelatine, and cryo-sections were cut under cryogenic conditions. Even though multiple blocks from six different patients were screened, no N protein was detected in the kidney. This might be due to the fact that, unlike the lung (analyzed both with FM and IEM), the tissue was degenerated and heavily damaged (data not shown), which is expected when materials are collected post-mortem. It remains unclear if the N protein accumulations were not preserved or were not present, and thus, we can only conclude that using post-mortem kidney tissue is more vulnerable to degeneration in comparison to lung tissue.

**TABLE 1:** Patient in description^*a*^

	Patient number	Biopsy/post-mortem	Age	Sex	Clinical presentation	Pathological findings	Remarks	COVID status
1	S20-47	PostM	75	F	ARDS/AKI		Plato study	Fatal COVID-19
2	S20-48	PostM	61	M	ARDS/AKI		Plato study; ultrastructure lost	Fatal COVID-19
3	S20-50	PostM	61	M	ARDS/AKI		Plato study; ultrastructure lost	Fatal COVID-19
4	S20-51	PostM	61	F	ARDS		Plato study; ultrastructure lost	Fatal COVID-19
5	S20-53	PostM	45	F	ARDS		Plato study; ultrastructure lost	Fatal COVID-19
6	S20-00060	PostM	66	M	ARDS/AKI		Plato study; ultrastructure reasonable	Fatal COVID-19
7	T21-40131	Biopsy	79	M	NS	FSGS and ATN	IF pos, IHC pos, PCR neg previously suffered from severe COVID-19; history of type 2 diabetes mellitus and hypertension. Biopsy taken 3 months after infection; proteinuria detected.	3 months post-COVID-19
8	T21-50496	Biopsy	57	F	NS	Collapsing FSGS and ATN	PCR neg; collapsing glomerulopathy tubular injury	5 days post-COVID −19
9	T21-5160	Biopsy	40	M	NS	Collapsing FSGS and ATN	Collapsing glomerulopathy, tubular damage after COVID	1 month post-COVID-19
10	T21-42029	Biopsy	50	M	AKI	TMA and ATN	Tubular injury during COVID; PCR negative	2 months post-COVID
11	T21-43254	Biopsy	73	M	AKI	ATN	Ultrastructure lost; collapsing glomerulopathy and tubular damage after COVID	1 month post-COVID
12	T22-40759	Biopsy	49	M	NS and AKI	Collapsing FSGS and ATN	Renal functional decline and nephrotic range proteinuria during hospital admission for COVID 19 pneumonia	During COVID-19 infection
13	T21-1150	Biopsy	61	F		BK polyomavirus nephropathy	2 1/2 years post transplantation	Control

^*a*^ ARDS, acute respiratory distress syndrome; AKI, acute kidney injury; NS, nephrotic syndrome; FSGS, focal and segmental glomerulosclerosis; ATN, acute tubular necrosis; TMA, thrombotic microangiopathy; PostM, post-mortem; IF pos, N protein present immuno-fluorescence; IHC pos, N protein present by immunohistochemistry as in Plato study (27).

### SARS-CoV-2 N protein was detected by FM in tubules of COVID-19 patient-derived kidney biopsies

**Fig 2:**
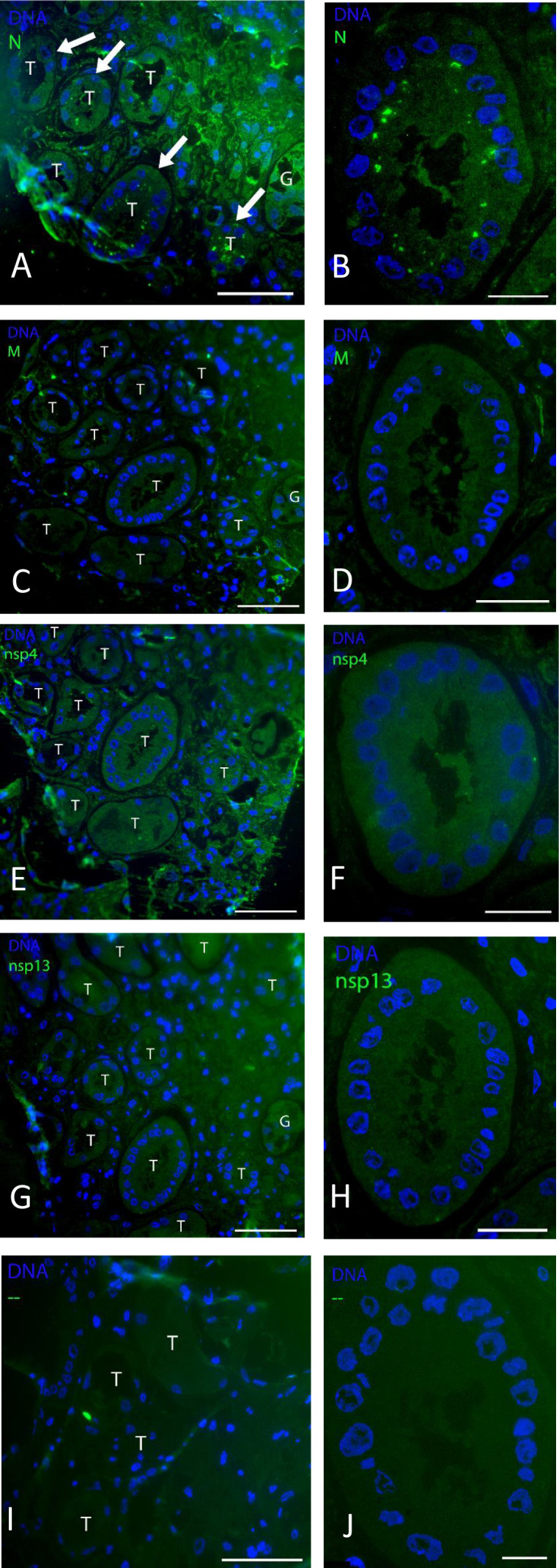
Fluorescence microscopy of viral proteins on kidney post-COVID-19 patient. On 300 nm, cryo-sections of kidney biopsy from post-COVID-19 patient 7 immunofluorescent labeling for: (A, B) N protein (in tubular epithelium highlighted with white arrows); (C, D) M protein; (E, F) nsp4; (G, H) nsp13. (I, J) As control, only secondary fluorescent antibody was used. Proteins of interest are labeled using Alexa 488 as a secondary antibody (green), the nuclei stained with Hoechst (blue). Scale bar left column is 60 µm and right column is 20 µm. T, tubular epithelium; G, glomerulus.

During, but also after, the COVID-19 pandemic, an increasing number of patients with AKI was observed. Biopsies from these patients were taken and processed as before (23) to screen for presence of viral proteins with FM using antibodies against SARS-CoV-2 proteins N and nsp4. In kidneys of seven different patients, the cortex was selected, and blocks were analyzed for the presence of viral proteins. In one patient (patient 7 in Table 1), N protein accumulations were evident in tubular epithelial cells (Fig. 2A and B). Several tubules were detected in which N protein had accumulated in multiple or even most of the cells. These N protein accumulations can be observed at the perinuclear region, at the apical luminal side, occasionally with multiple regions in one cell (Fig. 2A and B). Based on the arrangement of the nuclei and overall morphology, we propose that both distal and proximal tubules were positive for N protein. No accumulation in the renal interstitium nor in the glomeruli was evident.

As sequential or serial sections can be produced from the same region, multiple antibodies were tested for the presence of other viral proteins. However, no apical perinuclear accumulation of nsp4 and 13 nor M protein was detected in the regions with N protein containing tubules. M protein signal was irregularly detected in capillaries and in a few interstitial cells (Fig. 2C and D), and both for nsp4 and 13, merely individual spots were present (Fig. 2E through H). Also like in the uninfected Vero cells, some background is present for nsp4. As a control, the same immunofluorescence microscopy staining was performed without primary antibody (Fig. 2I and J) showing no staining.

Kidney biopsies from seven former COVID-19 patients experiencing kidney problems after infection were examined, and in a single patient, N protein accumulations were detected. To determine if these N clusters are protein or virus particles, a higher-resolution technique was necessary, and thus, immunoelectron microscopy was performed.

### SARS-CoV-2 N protein is present in tubules of COVID-19 patient-derived kidney tissue

**Fig 3:**
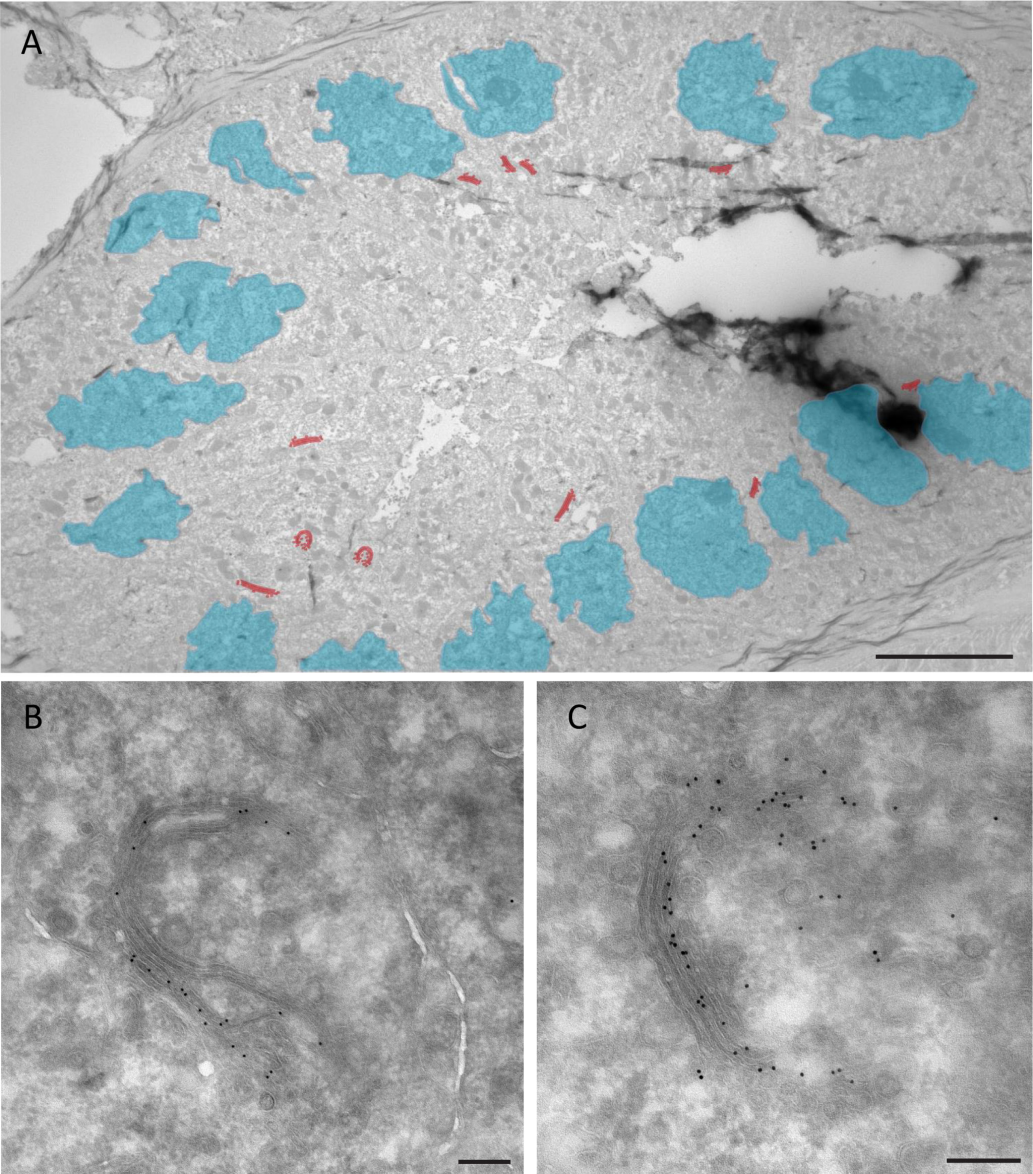
Electron microscopy of biopsy kidney tissue from COVID-19 patient. (A) Immunoelectron micrograph of kidney biopsy immunogold labeled for the N protein in overview with overlaid cartoon of nuclei (blue) and N protein-positive Golgi-like stacks (red). (B, C) Higher magnification demonstrates N protein decorated with 10 nm gold particles at membrane sheets of Golgi stacks. Scale bar represents 5 µm in (**A**) and 200 nm in (**B and C**).

Electron microscopy was employed to analyze tissue in high resolution and has previously been used on COVID-19-infected tissue to search for virus particles (21, 28). A valuable addition to this technique is immunogold labeling. This technique uses virus-specific antibodies detected by protein A conjugated to a 10 nm gold particle, which can be applied to precisely localize proteins on ultrathin (60–70 nm) LR white resin sections (28) or cryo-sections (23). After identification of N protein-positive kidney sections from patient 7 in FM, ultrathin sections were produced for further study using IEM with gold particles and N protein-specific antibody. An overview EM micrograph of a tubule shows (Fig. 3A and high resolution in Fig. S1) N protein accumulations denoted in red, which is similar to the localization done with FM, in the perinuclear regions denoted in blue. Some cells have multiple N protein-positive regions, a single region, or none. Higher magnification demonstrates that the immunogold labeling against N protein is present on membrane-rich clusters resembling the ultrastructure of Golgi stacks (Fig. 3B and C). In renal biopsies, five different tubules were found to be positive for N protein, and in these tubules, multiple cells accumulate N protein. Labeling was present on a series of stacked membranes and small vesicles pinching from or fusing with these membrane sheets. The morphology of the membrane sheets varies (Fig. S2A through F) from flat elongated (Fig. S2A) to curved and almost circular (Fig. S2B) even within a single tubular epithelium cell.

To understand the dynamics of the N protein on Golgi stacks of kidney epithelial cells, we determined if immunogold labeling was present on vesicles at *cis* face, on cristae, or at *trans* face for 15 different Golgi structures covered with N protein gold labels. In total, the sub-Golgi localization of 435 gold particles was determined, in which 20% was classified as present on *cis* face membranes and vesicles, 57% at the cristae, and 23% on the *trans* face membranes and vesicles. These findings indicate that the majority of N protein can be detected on the cristae of Golgi-like structures. It is important to note that only some of the vesicles surrounding the N protein-labeled Golgi stacks are indeed labeled, with at least one gold particle. From a total of 249 vesicles at the *cis* facing side of the Golgi stack, only 8% is labeled for N protein, and on *trans* face, from 225 vesicles, only 7% is labeled. As a control, the N protein label efficiency on virus particles in Vero cells was determined. In Vero cells infected for 24 h, 252 virus-like vesicles were scored, and 65% is labeled for N protein using the identical antibody and protocol as was used for the renal samples. Thus, immunogold labeling is less prominently present on vesicles surrounding Golgi stacks, compared to labeling on virus particles; thus, the question remains, are these vesicles virus particles? An important discriminating factor used to determine if small vesicles are virus particles is the size. The average size of the N protein-positive vesicles surrounding the Golgi stacks is 61 ± 13 nm (based on 36 immunogold-labeled vesicles), with no difference for *cis* or *trans* face (Fig. S2G). In Vero cells fixed and stained with an identical procedure, the average diameter of spherical virus particle size is 87 nm ± 17 nm and for oval-shaped particles 108 ± 27 nm (23). Notably, oval-shaped vesicles are rarely detected around the N-labeled Golgi stacks. This, together with the size difference and the sparse labeling, suggests that the vesicles detected surrounding the Golgi stacks in renal tissue are not virus particles.

### dsRNA is present in lipid-filled compartments but not in renal biopsies

**Fig 4:**
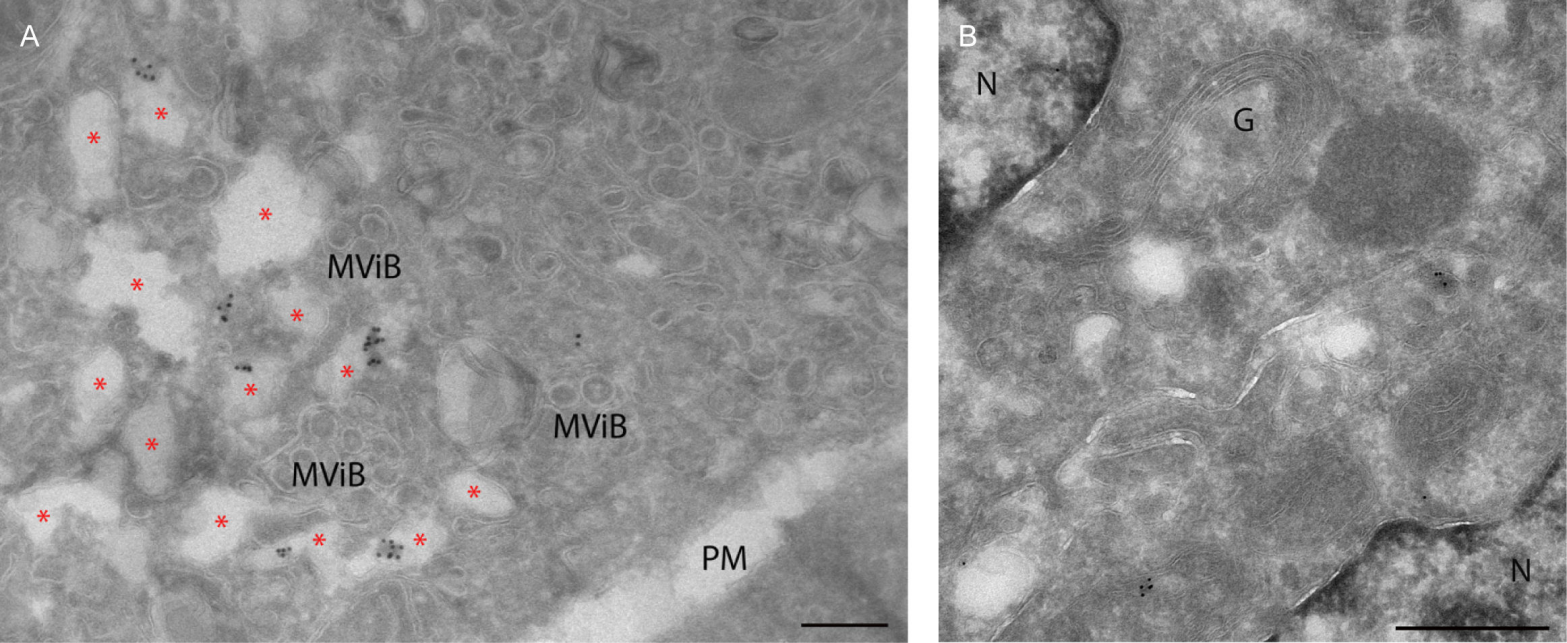
Electron microscopy of dsRNA immunogold labeling on a section of SARS-CoV-2-infected Vero cells and kidney from a COVID-19 patient. Immunogold labeling of a primary antibody detecting dsRNA followed by a secondary antibody conjugated with 10 nm gold particles on 60 nm thin cryo-sections of (A) SARS-CoV-2-infected Vero cells 24 h post-infection and (B) on sections of kidney biopsy of a post-COVID-19 patient. MViB,multivirus body; PM, plasma membrane; N, nucleus; G, Golgi stack; red asterisks, electron-lucent compartment. Scale bar represents 200 nm.

**Fig 5:**
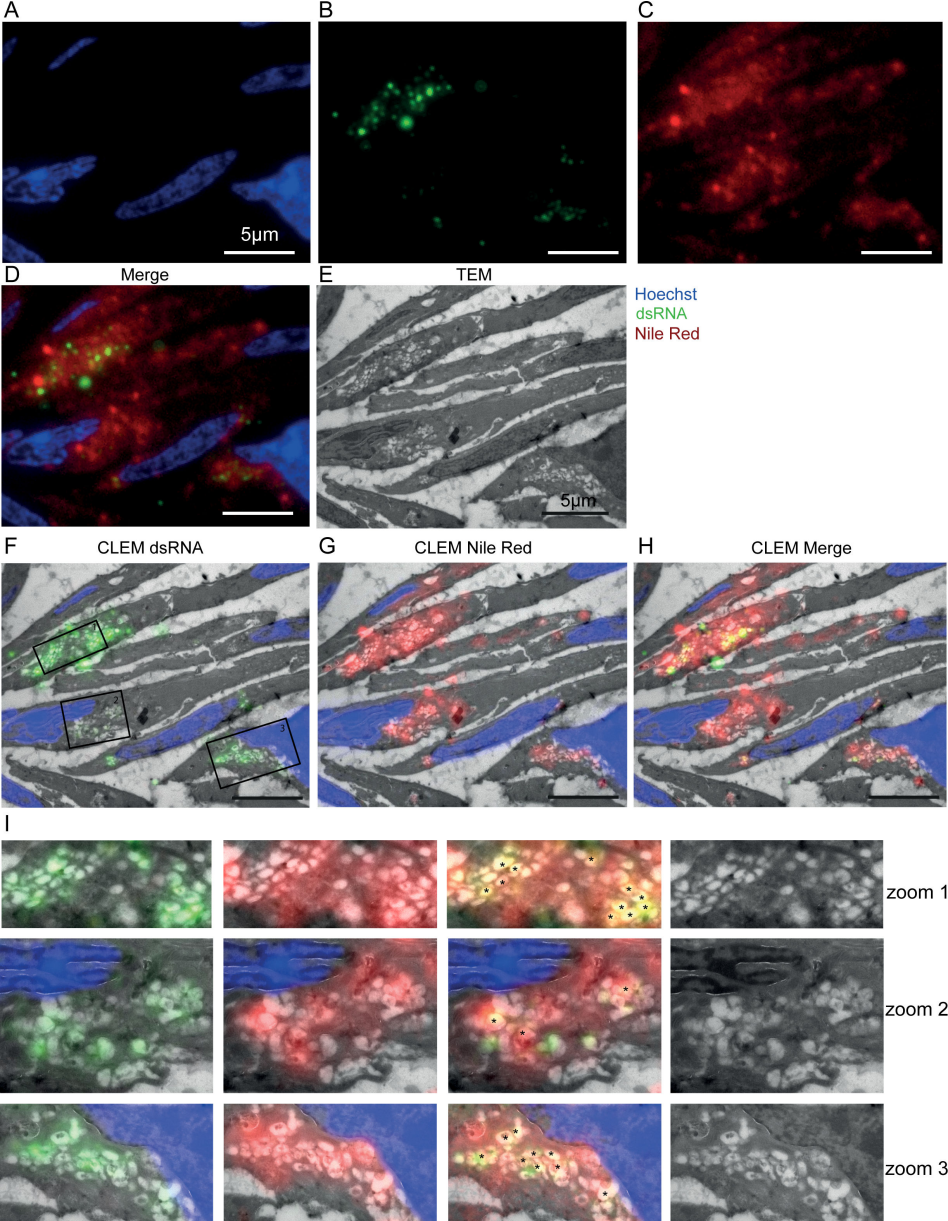
Combining light and electron microscopy demonstrates dsRNA in electron-lucent compartments in SARS-CoV-2-infected Vero cells. SARS-CoV-2-infected Vero cells sectioned for FM and stained with (A) Hoechst (blue) to identify nuclei; (B) primary antibody that detects dsRNA followed by secondary antibody with Alexa 488 (green) and (C) Nile red (red) to identify lipids. (D) A merged image co-localizing lipid and dsRNA (to yield yellow). (E )The identical section used for FM (**A–D**) was washed and stained with uranyl acetate and imaged using EM. (F–H) Electron micrograph overlaid with fluorescent images of nuclei (blue) combined with: (F) dsRNA signal (green) or (G) Nile red (red) to identify lipids; (H) the combined images of (F and G). The boxed areas (in F) are enlarged in I with zoom regions 1 (top), 2 (middle), and 3 (bottom) corresponding to color coding as in (**F–H**); black asterisks represent electron-lucent compartments indicating co-localization (yellow) of lipid and dsRNA. In all images, the scale bar represents 5 µm.

To determine if the renal epithelial cells with N protein produce viral RNA, we tested for the presence of dsRNA. Others have demonstrated that the presence of dsRNA indicates active viral production even though dsRNA is not a *bona fide* marker for viral RNA synthesis (24, 25, 29). Using FM on Vero cells, it already has been shown that dsRNA is present in punctate spots in the perinuclear region (Fig. 1). When Vero cells were prepared for and analyzed by IEM, similar regions were immunogold labeled (Fig. 4A). Even when aggregates in the antibody were removed by centrifugation and a dilution series was tested, immunogold labeling was clustered on electron-lucent compartments (Fig. 4A, marked with *) close to regions with virus-like particles and multivirus bodies (MViB). Electron-lucent compartments in *Mycobacterium tuberculosis* infections (30) and SARS-CoV-2-infected cells (23) were previously shown to be lipid-filled compartments. To determine if dsRNA is present in electron-lucent lipid-filled compartments, co-localization with fluorescent lipid stain Nile Red was performed on a section that was subsequently prepared for EM analysis to allow CLEM (Fig. 5; Fig. S3). The FM demonstrates at least part of the dsRNA (green) co-localizes (yellow) with lipid (red) in different cells. Not all cells are identical, and both dsRNA and lipid levels vary in fluorescent intensities. Higher magnification of the EM samples also shows that in some of the electron-lucent compartments, dsRNA and lipid (Fig. 5I marked with *) co-localize. As these compartments have a diameter of more than 300 nm (23) and as the thickness of these CLEM sections is 150 nm, it is possible that the clustered dsRNA is present in the previous or next section. Thus, the antibody against dsRNA is valid and can be used to detect dsRNA in renal biopsies by FM (data not shown) and EM (Fig. 4B). Besides some dispersed labeling in the cytosol, no specific localization of dsRNA was detected in kidney sections. Also, no dsRNA labeling was present on or near the Golgi stacks in renal tubules shown to be positive for the N protein.

### Extracellular vesicles were observed in kidney tubule lumen

**Fig 6:**
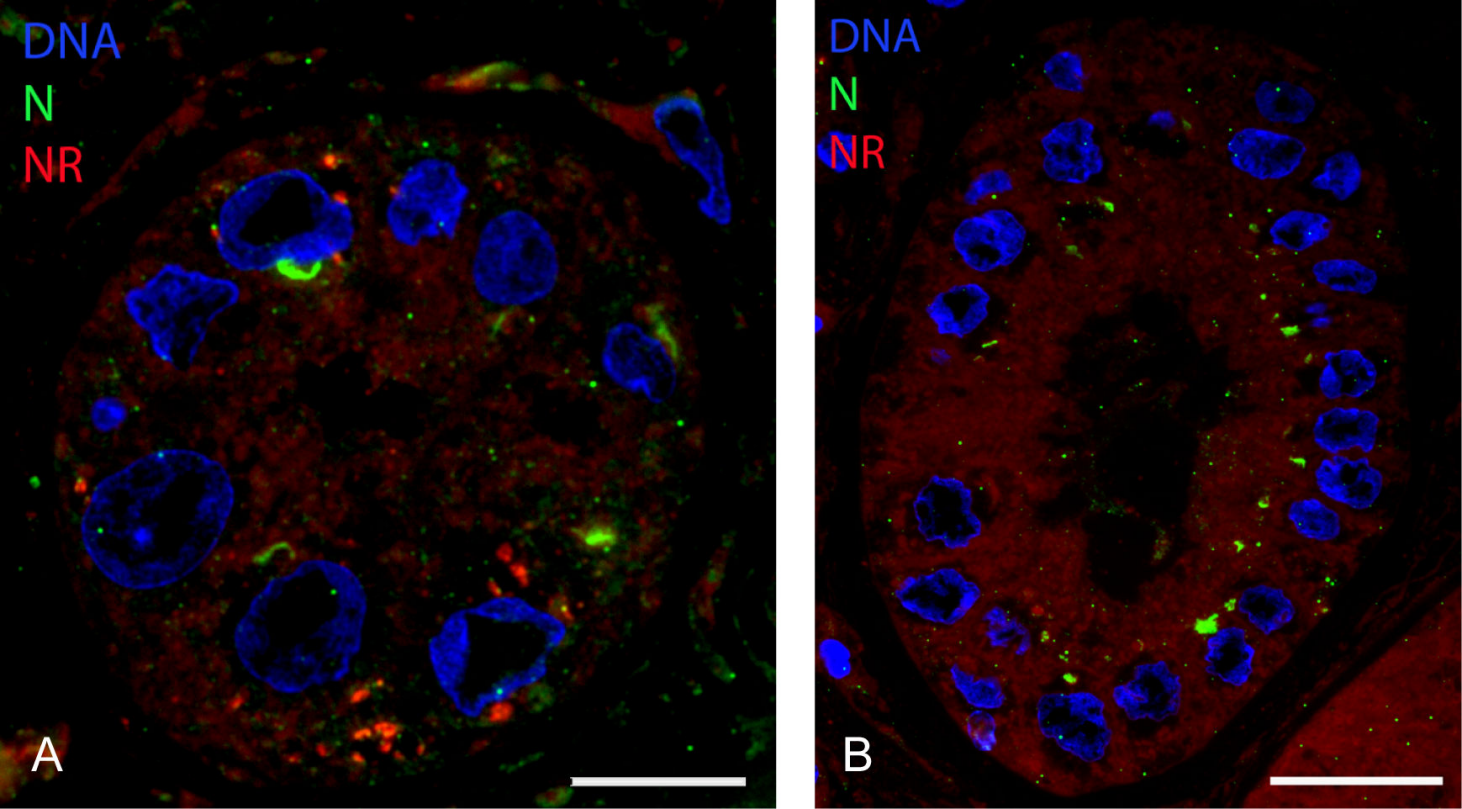
Fluorescence microscopy demonstration of lipids in a N protein-positive tubule from the kidney of a post-COVID-19 patient. (A) A semi-thin (300 nm) section of tubular endothelium from the kidney of a post-COVID-19 patient stained with Hoechst (blue) to identify nuclei, Alexa 488 (green) to identify N protein, and Nile red (red) to identify lipids. (B) Tubule stained similarly to A but without lipid accumulation (no red). Bar in (**A**) represents 10 µm and bar in (**B**) represents 20 µm.

**Fig 7:**
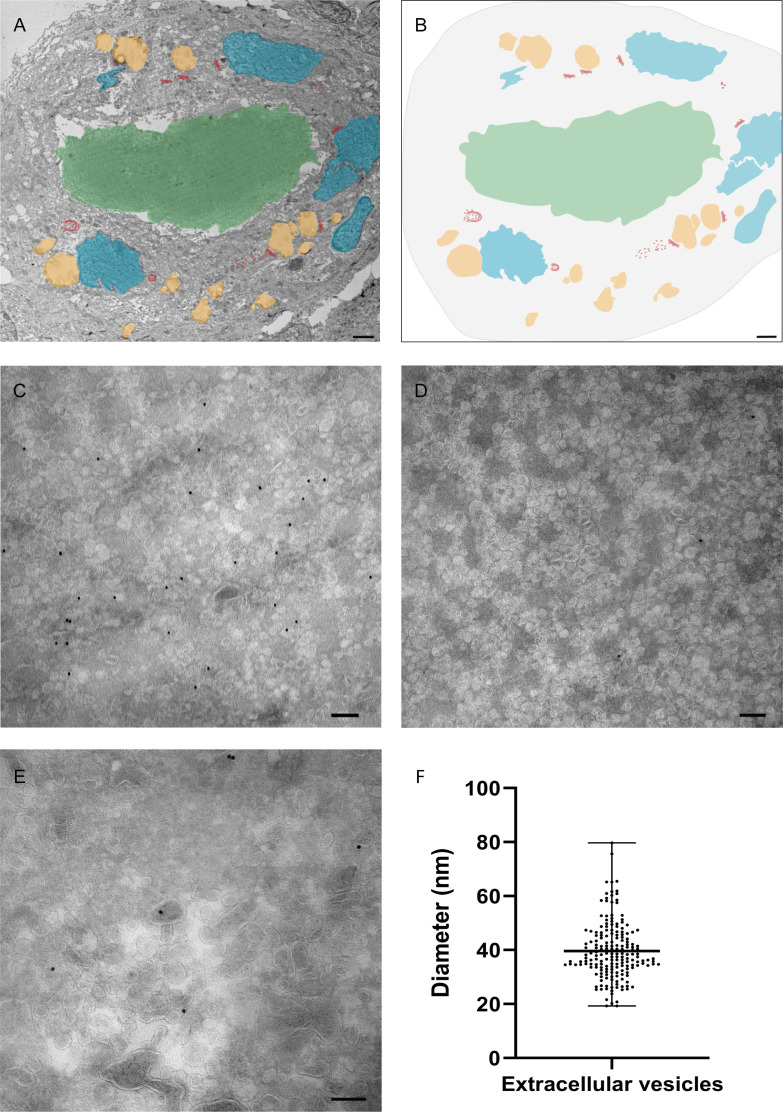
Electron micrograph of a tubule in kidney biopsy tissue from a post-COVID-19 patient in which the lumen is filled with vesicle-like structures. Electron micrograph of a 60 nm thin cryo-section of a renal tubule immunogold labeled for the viral N protein with an overlaid cartoon identifying various subcellular structures. (A) Electron micrograph combined with a cartoon (shown alone in B) showing nuclei in blue; electron-lucent compartments in orange; N protein-positive Golgi-like stacks (in red) and the vesicle-filled lumen (colored green). (B) The cartoon alone for identification of structures in (A). (C) High magnification of vesicle-filled tubular lumen with 10 nm immunogold labeling of exosome marker CD63 on vesicles, (D) low-level viral labeling dsRNA labeling on vesicles (similar to vesicles in C). (E) Viral N protein-labeled vesicles, one in the center resembling a SARS-CoV-2 virus particle [see reference (21) for ultrastructure of coronaviruses fixed by non-standard epoxy procedures; negative control sections were completely free of gold label]; (F) Average and standard deviation of diameter of 186 vesicles measured on five different electron micrographs from serial sections of one tubule, measured using ImageJ. Bar in (**A and B**) represents 2 µm and bar in (**C–E**) represents 100 nm.

As both in lung (23) and in Vero cells, electron-lucent lipid-filled compartments are induced by the SARS-CoV-2 virus, we examined renal tissue for lipid-filled compartments. Thus, on sections of renal biopsies, Nile red staining was combined with anti-N labeling (Fig. 6), and from five N-positive tubules examined, a single tubule had lipid accumulation (Fig. 6A). More detailed EM analysis demonstrated that in the one tubule with lipid accumulation, electron-lucent compartments can be detected (Fig. 7, orange coded). Based on the morphology and the absence of microvilli, this tubule was identified as a distal convoluted tubule. The lumen of this tubule is filled with small vesicles (Fig. 7C through E) averaging 40 nm in diameter, derived from measurements of 186 vesicles from four different sections (Fig. 7F). As they resembled exosomes, exosome marker anti-CD63 was tested, and it does indeed immunogold label the vesicles (Fig. 7C). No dsRNA was detected (Fig. 7D), and after labeling for N protein, an occasional gold particle was observed on a vesicle significantly larger than the urinary extracellular vesicles (Fig. 7E), which has a dense core.

## DISCUSSION

In this study, immunofluorescence microscopy was used to probe for viral proteins in renal biopsies of patients with fatal COVID-19 and patients with kidney failure. We were unsuccessful in finding viral proteins in post-mortem materials. As these tissues have been fixed in sub-optimal conditions, the ultrastructure might have been too damaged, which could have caused the loss of viral proteins. This is in contrast to the identically treated lung tissues of the same patients, where N protein accumulations were detected (23). Absence of N protein from these FM sections does not exclude presence of SARS-CoV-2 in these organs, as imaged blocks are small compared to the size of an intact kidney. The average single kidney volume is ~207 cm^3^ for men and ~157 cm^3^ for women (31), while the tissue blocks of only ~0.001 cm^2^ by 60–300 nm in thickness are subjected to microscopic analysis. Clearly, it is possible that if viral protein accumulation is not homogeneously distributed over the whole kidney cortex, this type of analysis could miss the regions where viral protein is accumulating. Light microscopy is more useful for screening larger samples. Indeed, we (27) and others (16, 32) previously detected N protein accumulations in the distal tubular epithelium of the kidney, and in some studies, three out of six patients were positive (11). Also, in our study, N protein accumulation is present in distal tubules in one out of seven renal biopsies of COVID patients with kidney failure. This part of the nephron is known to accumulate viral proteins or components that cannot be degraded. However, based on the localization in Golgi-like organelles and not in phagosomes or lysosome-like organelles, accumulation of non-degradable proteins is unlikely. While localization of N protein to Golgi stacks is unexpected, the Golgi is known to be affected by SARS-CoV-2 infection as it became fragmented in early stages of viral infection in Vero cells (26). However, in the same study, it was shown that overexpression of only the N protein did not cause rearrangements of the Golgi while other viral proteins did.

We screened for the presence of other viral proteins like the M protein, nsp3, 4, and 13, and dsRNA but detected none of these in the same region as the N protein. Absence of these viral nsps and dsRNA indicates no active viral replication in the observed kidney samples of these patients. The combination of numerous studies indicates that the expression of all nsp is required for replication of virus particles, reviewed by reference (33). In addition, in Vero cells actively producing virus, the N protein can be detected on vesicles surrounding the Golgi (23, 34, 35), but not at the cristae as detected in renal biopsy material. The M and S proteins have been localized to the Golgi stacks in infected cells (35), and S, E, and M proteins insert into the membrane of the endoplasmic reticulum (ER) and assemble into the endoplasmic ER-Golgi intermediate compartment (ERGIC) (36, 37). The subcellular localization of the N protein in renal biopsies, however, does not resemble the ERGIC and appears to differ from the localization observed in cultured cell systems. It is important to realize that the infection had been continuing for weeks, as patient 7 had suffered from COVID-19 already 3 months before the kidney problems manifested. To understand the cell biological mechanism of localizing N protein to Golgi stacks, analysis of prolonged infections for more than 24 h in a cell culture system are needed. However, it will be very difficult to mimic the long-term renal complications, even in Vero cells, as cell cultures will not survive such long-term infections. Thus, further patient material and animal model analysis at a subcellular level should be done to determine if the N protein accumulation in Golgi is a commonly occurring phenomenon.

Whether the N protein can be produced in tubular endothelium cells without the presence of dsRNA, nsp3, 4, and 13 is not known and remains to be further investigated. Proof of viral replication in human kidney is thus far not demonstrated and needs confirmation (6, 17, 38). In Vero cells, the presence of dsRNA in the lipid-filled compartments was detected with CLEM and suggests a role of lipid accumulation during replication. Indeed in Calu-3 cells, lipid accumulation enhanced the replication of various variants of SARS-CoV-2 (39). Also, we and others have previously demonstrated lipid accumulation in lung of COVID-19 patients (23, 40) and in monocytes derived from COVID-19 patients (41). Still, lipid accumulation in the N protein accumulating renal tubules was not consistently present, again suggesting that replication is not occurring at these late time points.

N protein accumulation could, however, have implications for the recurrence of kidney disease. It is the most abundantly produced protein during SARS-CoV-2 infections, and is very stable as was demonstrated based on the crystal structure (42). When remnants of the N protein remain in the kidney, it could have a continuing effect on the immune system. The N protein has been shown to interfere in innate immune response by modifying antiviral responses (43 – 45). Recently, presence of the N protein in the cytosol of cultured cells was shown to downregulate processing bodies [cytosolic RNA protein granules known to be involved in regulation of inflammatory cytokine production (46)]. Finally, N protein has been detected in urinary samples and has been correlated with disease severity (47). In our EM-based study, the only virus-like particles detected in kidney were the few in between the urinary extracellular vesicles. Infective SARS-CoV-2 virus has been isolated early in the SARS-CoV-2 pandemic from an autopsied kidney, under post-mortem conditions (48). It is possible that the virus is excreted from the body in between the small extracellular vesicles. Tubules filled with vesicles are not common in the kidney samples and were detected only incidentally in the current EM study. Others have reported slightly increased levels of urinary extracellular vesicles in COVID-19 patients (49). Still, the vesicle-filled lumens of renal endothelium is of interest. Performing electron microscopy on patient materials is challenging, but kidney biopsies can be of excellent quality and allow high-resolution morphology so that it is possible to discriminate viral protein accumulation from virus replication. The accumulation of N protein in the absence of other viral proteins described here needs to be further investigated to be able to understand the effects of the SARS-CoV-2 infection on acute kidney injury during post-COVID-19 complications.

## MATERIALS AND METHODS

### Origin of samples

For this study, tissue samples were obtained from seven (previously) hospitalized COVID-19 patients (Table 1). Autopsies and biopsies were performed at Amsterdam University Medical Centre (UMC) in The Netherlands, according to the declaration of Helsinki. SARS-CoV-2 infection before hospitalization was verified using quantitative real-time PCR.

Samples of uninfected and SARS-CoV-2-infected Vero E6 cells (Table S1) originated from the laboratory of Eric Snijder (23), and were treated, fixated, and embedded in gelatine as described in the paper.

### Reverse transcription and PCR for SARS-CoV-2

RNA extraction was performed using the Boom isolation method (50) with a total elution volume of 65 µL H_2_O. Equine arteritis virus was added before the RNA extraction as an internal extraction control. For cDNA synthesis, the SuperScript VILO cDNA Synthesis Kit (catalog no. 11754050, ThermoFisher Scientific, https://www.thermofisher.com) was used according to manufacturer’s instructions. cDNA synthesis was done on a 96-well Biometra thermal cycler for an initial step at 42°C for 30 min followed by 5 min at 85°C. PCR was done as described using the Light Cycler 480 (Roche) (51).

### Fixation and embedding of tissue samples for cryo-ultramicrotomy

Tissues were harvested, cut in small blocks, and immediately placed into fixative [0.1 M PHEM (PIPES, HEPES EGTA MgSO_4_ buffer pH 7.0), 2% paraformaldehyde, 0.5% glutaraldehyde]. Tissue blocks were cut with a razor blade into fragments of ~3 mm^3^ and further fixed in the same solution for 24 h. They were then transferred to storage solution (0.1 M PHEM, 0.5% paraformaldehyde) and stored at 4°C. Before embedding, samples were rinsed with phosphate-buffered saline (PBS) + 0.02 M glycine. Samples were cut further into segments of ~1 mm^3^ and embedded in gelatine using an increasing concentration of 2%, 5%, and 12% in 0.1 M phosphate buffer, 30 min per step. Excess gelatine was removed, and samples were stored overnight in 2.3 M sucrose at 4°C, while being rotated. Infiltrated samples were then transferred to microtome pins, snap-frozen in liquid nitrogen, and stored at −175°C until sectioning.

### Cryo-sectioning of gelatine-embedded samples

Tissue samples were sectioned using glass and diamond knives in a Leica Ultracut UC6 cryo-ultramicrotome. FM required semi-thin sections of 200–300 nm, sectioned at −80°C, which were transferred to a microscope slide using 2.3 M sucrose solution. For TEM, ultrathin sections of 60–70 nm are required, which were sectioned at −120°C, picked up in a droplet of methylcellulose and 1.15 M sucrose using a loop, and transferred to a Formvar-coated, 100 mesh hexagonal copper grid. Microscope slides and copper grids containing tissue sections were stored at 4°C until immunostaining.

### Immunofluorescent labeling and FM

Sections for FM were first washed three times using PBS + 0.02 M glycine, and then incubated for 1 h with antibody (Table S1 shows antibody dilution) diluted in PBS + 0.1% bovine serum albumin (BSA). Samples were washed with PBS + 0.02 M glycine, and subsequently incubated for 20 min in the dark with correct secondary antibody conjugated to Alexa 488, diluted in PBS + 0.1% BSA (Table S1). When performing CLEM, lipid (Nile Red) and nuclear (Hoechst) dyes, diluted in PBS + 0.1% BSA, were also added for 5 min (Table S1). When sections were only stained with lipid dyes, the first wash and incubation step with antibodies were skipped. After washing labeled sections with PBS + 0.02 M glycine, coverslips were mounted on the slide using Vectashield (Table S1), and it was subsequently sealed with nail polish. Slides were imaged using a Leica DM6 widefield microscope with a 100× oil objective.

### CLEM on semi-thin (150 nm) sections

The CLEM method was described in references 23 and 52. Briefly, from tissues fixed for immunoelectron microscopy, 100–300 nm sections were cut at −80°C and placed on a gold finder grid. These grids were washed on droplets of PBS + 0.02M glycine (Merck, K27662101) for three times, 5 min each wash. Thereafter, the grids were incubated with primary antibody mouse αdsRNA (diluted and centrifuged for 20 min at 10,950 × *g*) in 0.1% BSA and washed with PBS + 0.02M glycine for three times, 5 min each. After that, grids were incubated for 20 min with secondary goat antibody made against mouse serum (goatαmouse) conjugated to Alexa 488 (Mol. Probes, A32731), followed by 20-min incubation in Nile red (1 mg/mL in ethanol, Sigma, 72485) and Hoechst 33342 (ThermoFisher, H3570, 10 mg/mL diluted 1:100 in water). They were then washed with PBS three times for 5 min and mounted in between a glass slide and a coverslip using a droplet of Vectashield (Vector Scientific, H-1000). Imaging was performed using a Leica DM6 widefield microscope with a 100× oil objective. After widefield imaging, the coverslip was removed from the glass slide by pipetting PBS in between the coverslip and the glass slide. Vectashield was washed from the grid by incubation in water at 37°C for six times, 10 min each wash. Thereafter, the grids were contrasted for EM with 3.5% uranyl acetate in 2% methylcellulose (Sigma, M6385-250G) for 5 min, and the excess was blotted from the grid by gently moving along on a filter paper. After drying for at least 20 min, regions of interest were imaged using a FEI Tecnai 120kV TEM (ThermoFisher). Low magnification images were analyzed using ImageJ Fiji (https://imagej.net/software/fiji/), and the correlation was performed using ICY software with eC-CLEM [ec-CLEM | – Open Source Image Processing Software (bioimageanalysis.org)] plugin.

### Immunogold labeling of tissue sections and TEM

Copper grids containing ultrathin cryo-sections were first incubated for 30 min at 37°C on plates containing 2% gelatine in 0.1 M phosphate buffer. Grids were taken from the gelatine solution, washed in PBS + 0.02 M glycine, and blocked with PBS + 0.02M glycine +1% BSA. Sections were incubated for 60 min with primary antibodies αN protein, αdsRNA (centrifuged for 20 min at 10,950 × *g*), αNsp4, αM protein, αLAMP-1, or αACE2 (for information on antibodies, see Table S1.) in PBS + 1% BSA, and then washed with PBS + 0.02M glycine. Grids incubated with monoclonal mouse antibodies were then incubated for 20 min with rabbit anti-mouse bridging antibody diluted in PBS + 1% BSA; washed six times for 3 min with PBS + 0.02M glycine, and blocked with PBS + 0.02M glycine +0.1% BSA. All grids were incubated for 20 min with 10 nm colloidal gold (diluted 1:60) and subsequently washed six times for 3 min each with PBS + 0.02M glycine. Formed antibody-immunoglobulin complexes were fixed by incubating sections with PBS + 1% glutaraldehyde for 5 min. After washing fixed samples 10 times for 2 min on a droplet of water, sections were contrasted with uranyl acetate in 2% methylcellulose for 5 min. Blotting paper was used to remove excess uranyl acetate solution, and grids were transferred to a grid box, stored at room temperature. Sections were imaged with an FEI Tecnai 120 kV TEM, using either a Veleta or a Xarosa (EMSIS) camera.

### Measurements of vesicle size

For the average diameter of vesicles surrounding Golgi stacks, 16 electron micrographs with individual Golgi stacks immunogold labeled for N protein were used. At the *cis* face, 20 vesicles and, at the *trans* face, 16 vesicles with N protein labeling were measured. For the analysis of the diameter of inter-luminal vesicles, five electron micrographs were used, and 186 vesicles were measured. Vesicle diameter was measured on images calibrated for correct magnification using Fiji (ImageJ). Statistical analysis to determine average and standard deviation, excel, and R were used.

## SUPPLEMENTAL MATERIAL

Supplemental file 1 spectrum.03029-23-s0001.docx Supplemental material.

ASM does not own the copyrights to Supplemental Material that may be linked to, or accessed through, an article. The authors have granted ASM a non-exclusive, world-wide license to publish the Supplemental Material files. Please contact the corresponding author directly for reuse.
