# Supplementary material for: Nucleocapsid protein accumulates in renal tubular epithelium of a post-COVID-19 patient
Source: Microbiol Spectr. 2023 Nov 17;11(6):e03029-23. doi: 10.1128/spectrum.03029-23 (PMC10715010; doi:10.1128/spectrum.03029-23)
Supplement: Supplemental file 1 — Supplemental material. [file spectrum.03029-23-s0001.docx]

**Supplemental Figures**

All Figures with interactive mode are available at:

[https://create.figlinq.com/~n.n.vanderwel/163/?share_key=LPU8yj62EKvCKxAkEO5pEs](https://eur04.safelinks.protection.outlook.com/?url=https%3A%2F%2Fcreate.figlinq.com%2F~n.n.vanderwel%2F163%2F%3Fshare_key%3DLPU8yj62EKvCKxAkEO5pEs&data=05%7C01%7Cs.vanderniet1%40amsterdamumc.nl%7Ca180277d2a98455fca2f08db5de10bb5%7C68dfab1a11bb4cc6beb528d756984fb6%7C0%7C0%7C638206993357017242%7CUnknown%7CTWFpbGZsb3d8eyJWIjoiMC4wLjAwMDAiLCJQIjoiV2luMzIiLCJBTiI6Ik1haWwiLCJXVCI6Mn0%3D%7C3000%7C%7C%7C&sdata=FtoXrlGAt4hUI48vyIUHjC9gkFE4RHPXQ%2BTNsl3WR5I%3D&reserved=0)

**
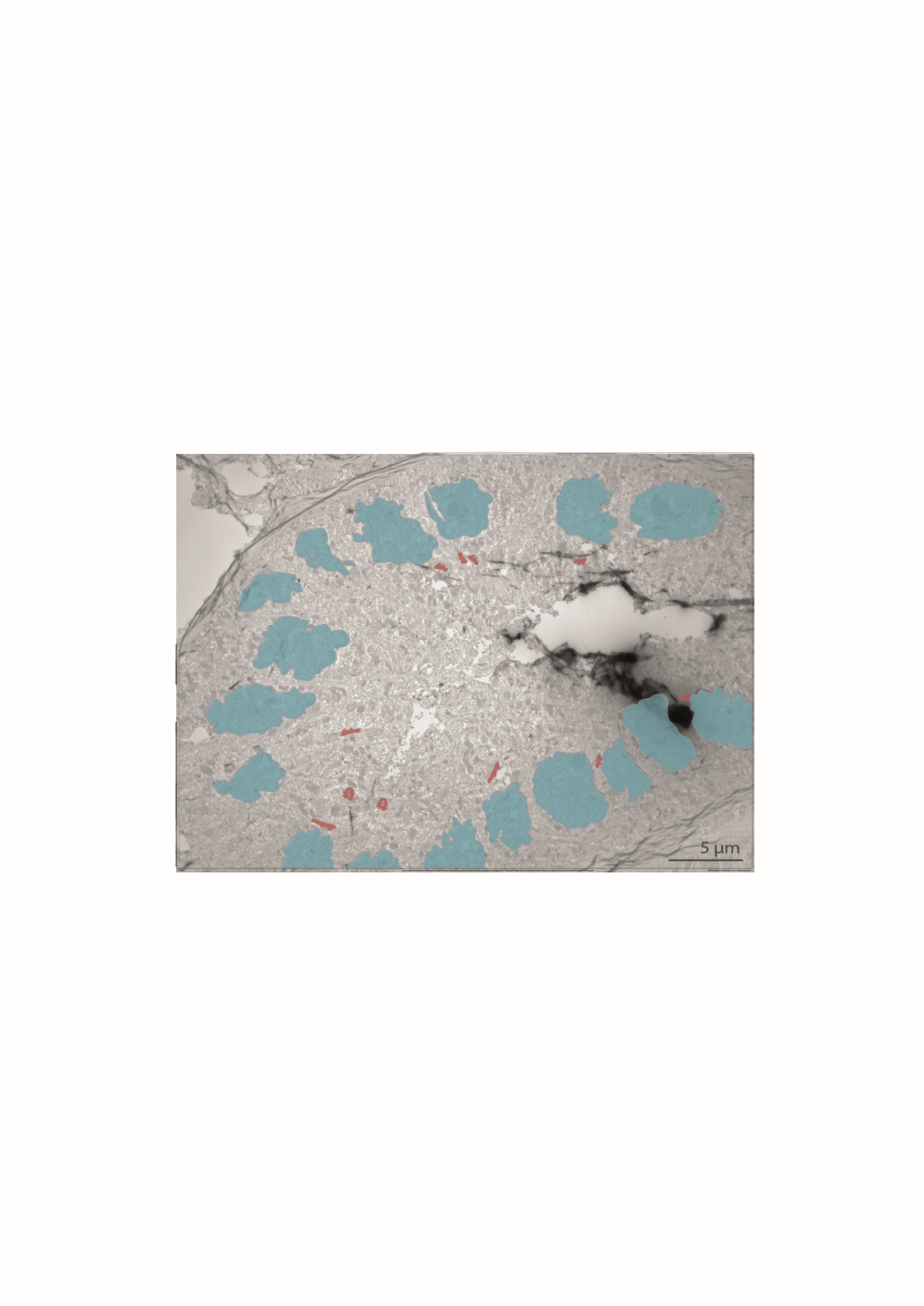
**

**Figure S1: Electron Microscopy of biopsy kidney tissue from COVID-19 patients.** Interactive image with first layer, immuno-electron micrograph of kidney biopsy immuno-gold labelled for the N-protein in overview. In layer 2 the electron micrograph is overlaid with a color-coded cartoon to identify nuclei (blue), N-protein positive Golgi-like stacks (red). Layer 3 is cartoon only. Bar represents 5 µm.


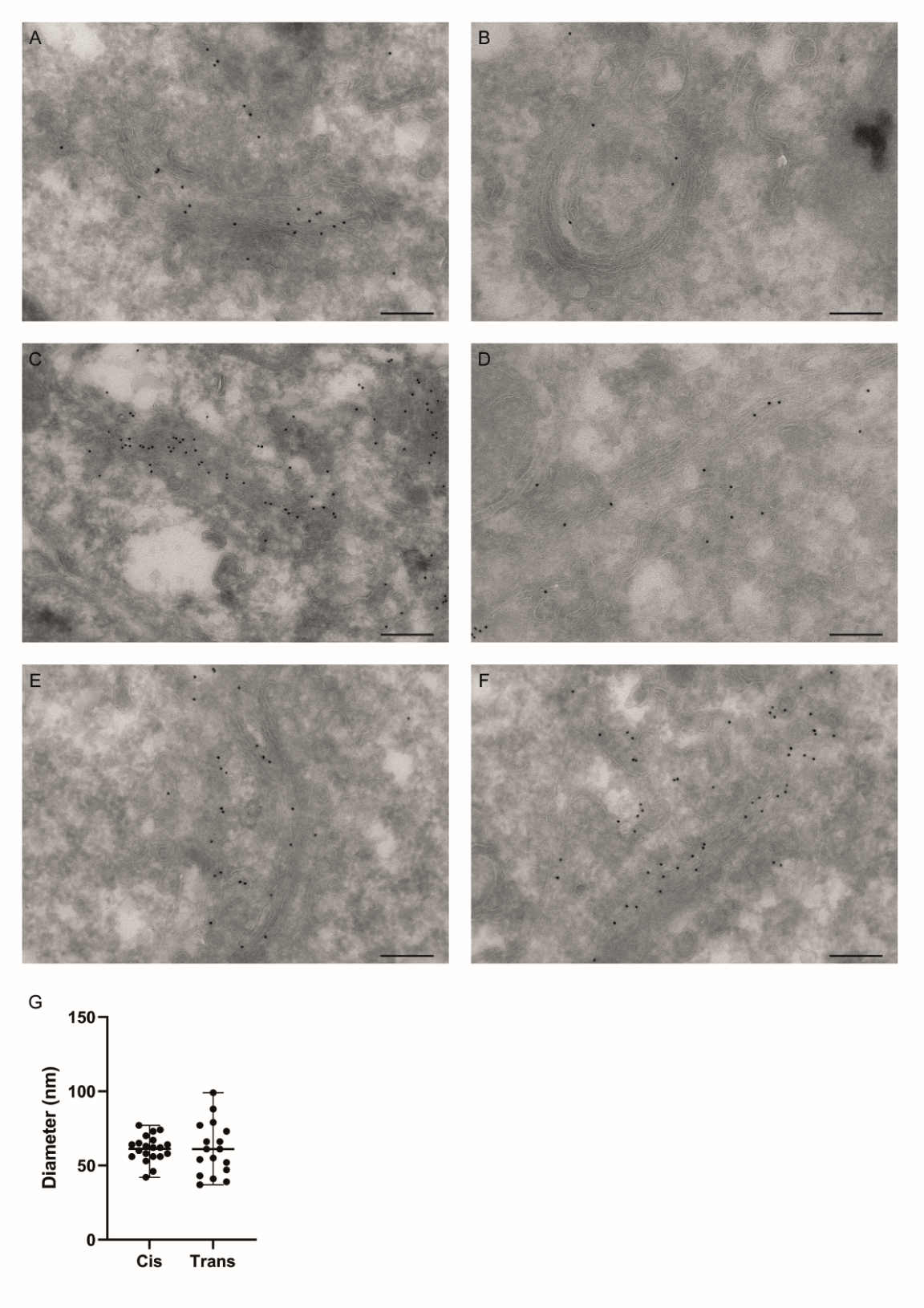


**Figure S2: Golgi stacks from biopsy kidney tissue from long COVID-19 patient with N-protein. A-F.** Representative N-protein positive immuno-gold (10 nm) labelled membrane rich clusters which resemble the ultrastructure of Golgi stacks. **G.** Vesicles of average diameter surrounding the Golgi at cis face and trans face, based on 16 micrographs of N-protein labelled Golgi stacks as demonstrated in **A-F**. Bars represents 200 nm.


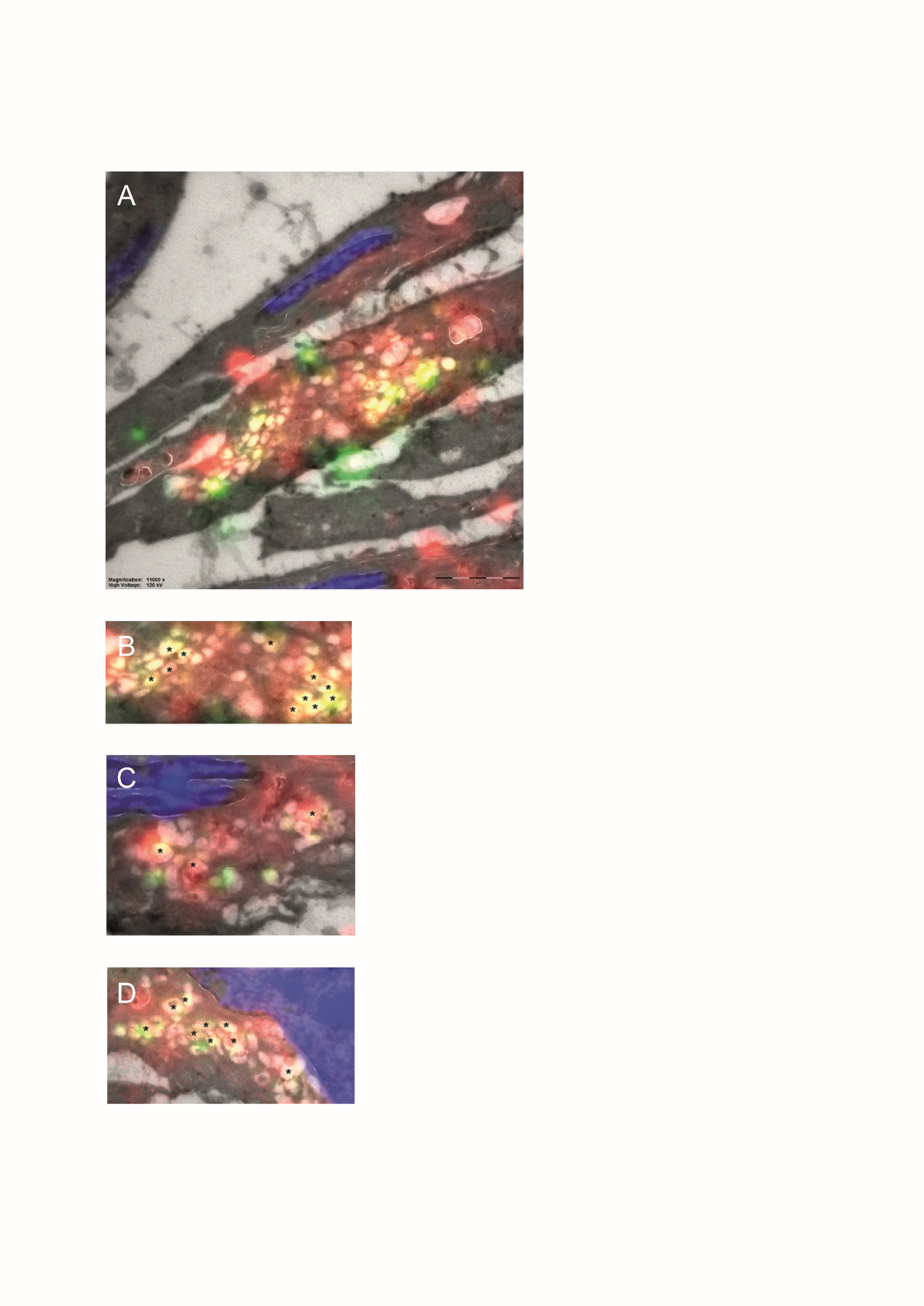


**Figure S3: CLEM demonstrates dsRNA in electron-lucent compartments in SARS-CoV-2 infected Vero cells. A-D.** Interactive images of 24 hours SARS-CoV-2 infected Vero cells sectioned for FM and stained with Hoechst (blue) to identify nuclei, Nile red (red) to identify lipids, primary antibody detecting dsRNA followed by secondary antibody with Alexa 488 (green). **A**. The FM image is merged with the EM image from Figure 5B showing co-localization (yellow) of dsRNA with lipid. **B**. Zoomed region 1 of Figure 5, **C**. zoomed region 2, **D**. zoomed region 3; asterisks mark yellow color co-localization.

Supplemental table

**Table S1** List of materials

| Reagent or resource | Final dilution | Source | Identifier |
| --- | --- | --- | --- |
| Antibodies |  |  |  |
| Mouse monoclonal anti-N | 1:500 | SinoBiological | Cat. no. 40143-MM05 |
| Mouse monoclonal anti-dsRNA J2 | 1:500 | SCICONS | Cat. no. 10010200 |
| Mouse monoclonal anti-ACE2 | 1:500 | Thermo Fisher | Cat. no. MA5-32307 |
| Rabbit polyclonal anti-LAMP1 | 1:50 | Biolegend | Cat. no. H4A3 |
| Mouse Human anti-CD63 | 1:100 | Santa Cruz | Cat. no. MX 49.129.5 |
| Rabbit polyclonal anti-Nsp4 | 1:100 | Kind gift from Snijder laboratory | Leiden University Medical Centre |
| Rabbit polyclonal anti-M | 1:100 | Kind gift from Snijder laboratory | Leiden University Medical Centre |
| Rabbit Bridging anti-mouse | 1:200 | DAKO | Cat. no. Z0259; RRID20007985 |
| Goat anti-mouse Alexa 488 | 1:500 | Life technologies | Cat. no. A21242; RRID1345066 |
| Protein A conjugated to 10-nm gold | 1:25 | Utrecht university | https://cellbiology-utrecht.nl/products.html |
| Chemicals |  |  |  |
| Nile red | 1:25 | Sigma-Aldrich | 72485 |
| Hoechst 33342 | 1:100 | Thermo Fisher | H3570 |
| Phosphate-buffered saline (PBS) | 1x | Gibco | 18912-014 |
| Glycine | 0.02M | Merck | K27662101 |
| Gelatine | 2% and 12% | Sigma-Aldrich | G2500-500G; CAS9000-70-8 |
| Methylcellulose (MC) | 2% | Sigma-Aldrich | M6385-250G; CAS9004-67-5 |
| Bovine serum albumin (BSA) | 10% | Sigma-Aldrich | A4503-50G; CAS9048-46-8 |
| Vectashield antifade mounting medium | Undiluted | Vector Laboratories | H-1000 |
| Uranyl acetate (UA) in MC | 0.35% | EMS | 22400 |
| Virus strain: nCoV-2019/Melb-1 | X | Kind gift from Snijder laboratory | X |
| Experimental model: cell line Vero E6 cells | X | Kind gift from Snijder laboratory | X |
